# Increased Serum Interleukin-34 Levels Are Related to the Presence and Severity of Cardiac Dysfunction in Patients With Ischemic Cardiomyopathy

**DOI:** 10.3389/fphys.2018.00904

**Published:** 2018-07-11

**Authors:** Rui Xi, Qin Fan, Xiaoxiang Yan, Hang Zhang, Hongyang Xie, Gang Gu, Yan Xu, Fang Wang, Rong Tao

**Affiliations:** ^1^Department of Cardiology, Ruijin Hospital, Shanghai Jiao Tong University School of Medicine, Shanghai, China; ^2^Institute of Cardiovascular Diseases, Shanghai Jiao Tong University School of Medicine, Shanghai, China

**Keywords:** interleukin 34, heart failure, ischemic cardiomyopathy, coronary artery disease, inflammation

## Abstract

**Background:** Several inflammatory factors have been demonstrated with diagnostic or prognostic value in patients with ischemic cardiomyopathy (ICM). Interleukin 34 (IL-34), an additional ligand of colony stimulating factor-1 receptor (CSF-1R), has been identified as a biomarker of coronary artery disease (CAD) and chronic kidney disease (CKD). However, the potential effect of IL-34 in ICM remains unknown.

**Methods:** Serum IL-34 levels were detected in 360 subjects with ICM and in 465 subjects without ICM; the latter group included 233 controls without CAD and 232 patients with CAD and normal cardiac function. Uni- and multivariable logistic regression analyses were conducted to analyze the relationship between IL-34 and ICM.

**Results:** IL-34 levels were significantly increased in patients with ICM compared with both groups of subjects without ICM (122.52 ± 115.30 vs. 95.02 ± 101.43 vs. 82.57 ± 84.24 pg/ml, respectively; *P* < 0.001). Moreover, serum IL-34 level was significantly positively correlated to NT-proBNP level (*r* = 0.223, *P* < 0.001), left ventricular end diastolic diameter and New York Heart Association (NYHA) functional class, indicating that a higher IL-34 level reflects more severe heart failure (HF). Multivariable regression analyses revealed that IL-34 was remarkably associated with the presence and severity of ICM after adjusting for age, sex, conventional risk factors as well as medication [odds ratio (OR): 1.501, 95% confidence interval (CI): 1.249–1.803, *P* < 0.001, per SD increase]. The predictive value of IL-34 value remained significant in patients already diagnosed with CAD.

**Conclusion:** Increased IL-34 levels are relevant to the presence and severity of ischemic HF in all subjects and in patients with CAD. IL-34 may be used as a novel clinical biomarker of ICM with predictive value.

## Introduction

Ischemic cardiomyopathy (ICM), also known as ischemic heart failure (HF), is a myocardial disease secondary to severe coronary atherosclerotic lesions, which leads to chronic, long-term myocardial ischemia and hypoxia, myocardial cell damage, apoptosis, necrosis, and myocardial fibrosis, resulting in cardiac structural changes and decreased cardiac function (Rydlewska et al., 2011). It is the leading cause of HF (Roger et al., 2012). In spite of the recent progress achieved in its management, ICM morbidity and mortality rates still continue to increase, causing serious social economic burden (Benjamin et al., 2017). Coronary artery stenosis or occlusive disease is the primary cause of ICM, and it could aggravate the process of ischemic HF in turn (Go et al., 2006). Inflammation and fibrosis have been found to play critical roles in the initiation and progression of atherosclerosis, myocardial ischemic injury and HF (Epelman et al., 2015). Several inflammation-associated cytokines such as IL-6, IL-1β, TNF-α and sST2 showed predictive value in the pathogenesis, risk stratification, diagnosis, and prognosis of HF (Mann, 2015).

Interleukin-34 (IL-34) is a cytokine that was first determined as an additional ligand of Colony Stimulating Factor-1 Receptor (CSF-1R); it is expressed in several organs, including the heart, lung, kidney, liver, brain, and spleen (Lin et al., 2008), and acts as a major factor regulating proliferation, differentiation, and survival of mononuclear phagocytes which include macrophages, monocytes as well as osteoclasts (Nakamichi et al., 2013). Additionally, it can induce the expression and secretion of some pro-inflammatory cytokines (Eda et al., 2010), participating in the inflammatory process of different diseases, such as rheumatoid arthritis (Chemel et al., 2012) and inflammatory bowel disease (Zwicker et al., 2015). Other recent evidence demonstrated a relationship between IL-34 and the progress of fibrosis via increased expression of galectin-3 (Preisser et al., 2014), which is an important biomarker of HF, suggesting its role in mediating cardiac remodeling in ICM. Previous studies also showed that higher serum IL-34 levels were significantly related to more severe coronary artery disease (CAD) in patients with normal cardiac function (Li et al., 2012) as well as in patients with chronic HF (Fan et al., 2016). It might contribute to ischemic myocardial injury and atherosclerosis mainly by regulating the migration and differentiation of macrophages and monocytes (Swirski et al., 2009; Robbins et al., 2012).

However, the specific role and clinical significance of IL-34 in the development of ischemic HF is less well established. Therefore, in the present study our aim was to explore the clinical significance of IL-34 in ICM, expecting to find novel targets for intervention or treatment of this disease.

## Materials and Methods

### Patient Population and Study Design

Totally 825 Chinese patients with suspected CAD who underwent selective coronary artery angiography were consecutively enrolled in this cross-sectional observational study. The exclusion criteria consist of acute coronary syndrome or stroke within 4 weeks before assessment, or significant concomitant diseases including cancer, serious infections, or autoimmune diseases. All included subjects were aged 18 years or above.

Chronic HF was defined as using diuretics for ≥3 months, hospitalization because of HF at least once within a year at the inception of the study, also plasma levels of amino-terminal pro-brain natriuretic peptide (NT-proBNP) >300 pg/mL for subjects in sinus rhythm or >900 pg/mL for atrial fibrillation patients at baseline, and cardiac structural changes including the left ventricle hypertrophy and/or left atrial enlargement proved by echocardiography. All those patients should have symptoms and signs of HF at baseline. Patients with HF who met one of the following conditions: previous history of myocardial infarction or revascularization, left main or left anterior descending proximal coronary branch stenosis, along with double or triple CAD (defined as ≥ 75% reduction in luminal diameter), were included in the ICM group according to previous studies (Felker et al., 2002; Sardi et al., 2012). Patients who met the above criteria, but had no HF, served as the CAD group, while those without coronary artery stenosis were included in the control group.

The study was approved by the human research committee of Shanghai Jiao Tong University School of Medicine affiliated Ruijin Hospital. It was conducted in accordance with the ethical guidelines of the 1975 Declaration of Helsinki. Before data collection at baseline, each subject enrolled in the study provided signed informed consent with well understanding.

### Data Collection and Measurements

Data were recorded at baseline via face-to-face interviews and included information regarding medical history, results of physical examination, details regarding concomitant diseases and major combined medications, as well as the values of routine laboratory tests which were listed in **Table 1**. For each subject, echocardiography was conducted on the same day (or within a week) when the peripheral blood samples were obtained.

**Table 1:** Baseline characteristics of all subjects.

	Con (*n* = 233)	CAD (*n* = 232)	ICM (*n* = 360)	*P*-value
Age (y)	60.06 ± 10.22	61.36 ± 11.42	65.67 ± 11.41	<0.001
Male, gender	126 (54.10)	186 (80.20)	303 (84.20)	<0.001
Smoker	60 (25.80)	100 (43.10)	179 (49.70)	<0.001
Alcohol use	34 (14.60)	38 (16.40)	83 (23.10)	0.020
BMI (kg/m^2^)	24.52 ± 3.44	25.34 ± 3.49	24.46 ± 3.32	0.005
Systolic blood pressure (mmHg)	133.71 ± 17.24	129.42 ± 20.11	127.62 ± 22.37	0.002
Diastolic blood pressure (mmHg)	76.62 ± 11.43	74.56 ± 11.56	75.15 ± 13.20	0.170
Heart rate (beats/min)	76.14 ± 10.89	76.26 ± 11.53	79.43 ± 14.69	0.002
**Medical history**		
• Diabetes mellitus	41 (17.60)	64 (27.60)	140 (38.90)	<0.001
• Hypertension	134 (57.50)	157 (67.70)	252 (70.00)	0.006
• Dyslipidemia	93 (39.90)	71 (30.60)	124 (34.40)	0.106
• CKD	11 (4.70)	20 (8.60)	107 (29.70)	<0.001
• OMI	0 (0)	126 (54.30)	256 (71.10)	<0.001
• Stroke	11 (4.70)	31 (13.40)	66 (18.30)	<0.001
• COPD	5 (2.10)	3 (1.30)	27 (7.50)	<0.001
• AF	18 (7.70)	9 (3.90)	46 (12.80)	0.001
**Echocardiography**		
• LVEF (%)	66.58 ± 4.78	63.59 ± 5.81	44.62 ± 8.96	<0.001
• LVEDd (mm)	47.68 ± 3.80	49.50 ± 4.54	58.81 ± 7.00	<0.001
• LVESd (mm)	29.94 ± 3.34	32.15 ± 4.40	45.35 ± 7.82	<0.001
• LAd (mm)	37.99 ± 4.37	38.51 ± 3.51	43.14 ± 4.94	<0.001
**Lab. Examination**		
WBC (^∗^10^9^/L)	6.06 ± 1.68	7.19 ± 2.45	7.51 ± 2.42	<0.001
Hemoglobin (g/L)	134.99 ± 14.31	135.47 ± 12.54	129.76 ± 17.18	<0.001
Platelets (^∗^10^9^/L)	196.98 ± 60.89	192.44 ± 53.63	185.58 ± 63.73	<0.001
Fasting glucose (mmol/L)	5.20 ± 1.21	5.84 ± 2.06	6.02 ± 2.01	<0.001
HbA1c (%)	5.96 ± 0.90	6.37 ± 1.31	6.55 ± 1.28	<0.001
Alb (g/L)	39.00 ± 3.20	37.25 ± 3.18	35.31 ± 4.83	<0.001
BUN (mmol/L)	5.58 ± 1.67	5.46 ± 1.41	6.92 ± 3.39	< 0.001
Creatinine (μmol/L)	76.46 ± 15.68	83.53 ± 18.79	101.15 ± 71.50	<0.001
Uric acid (μmol/L)	322.89 ± 85.34	342.97 ± 86.26	365.44 ± 123.65	<0.001
eGFR_MDRD_ (ml/min/1.73m^2^)	81.99 ± 15.79	80.68 ± 17.65	72.72 ± 23.01	<0.001
Cystatin C (mg/L)	1.03 ± 0.22	1.07 ± 0.25	1.34 ± 0.81	<0.001
Total cholesterol (mmol/L)	3.98 ± 1.03	4.27 ± 1.01	3.97 ± 1.04	0.001
Triglyceride (mmol/L)	1.68 ± 1.05	1.75 ± 1.15	1.46 ± 0.70	0.001
LDL-C (mmol/L)	2.38 ± 0.90	2.54 ± 0.82	2.41 ± 0.87	0.089
HDL-C (mmol/L)	1.15 ± 0.33	1.01 ± 0.29	0.97 ± 0.25	<0.001
hsCRP (mg/L)	2.41 ± 7.39	8.03 ± 19.73	22.78 ± 48.64	<0.001
NT-proBNP (pg/ml)	122.47 ± 199.96	252.27 ± 254.67	3179.51 ± 4505.83	<0.001
Troponin I (ng/mL)	0.01 ± 0.02	0.38 ± 0.84	0.49 ± 0.96	<0.001
IL-34 (pg/ml)	82.57 ± 84.24	95.02 ± 101.43	122.52 ± 115.30	<0.001
**Medications**		
• ACEI/ARB	96 (41.20)	159 (68.50)	263 (73.10)	<0.001
• β-blocker	125 (53.60)	191 (82.30)	306 (85.00)	<0.001
• Antiplatelet drugs	170 (73.00)	229 (98.70)	348 (96.70)	<0.001
• Nitrates	21 (9.00)	78 (33.60)	176 (48.90)	<0.001
• Statins	114 (48.90)	207 (89.20)	319 (88.60)	<0.001
• CCB	53 (22.70)	56 (24.10)	52 (14.40)	<0.001
• Hypoglycemic drugs	25 (10.70)	41 (17.70)	72 (20.00)	0.011


*Data are presented as mean ± SD or n (%). ACEI, angiotensin-converting enzyme inhibitor; AF, atrial fibrillation; ARB, angiotensin receptor blocker; BMI, body mass index; BUN, blood urea nitrogen; CCB, calcium channel blocker CKD, chronic kidney disease; COPD, chronic obstructive pulmonary disease; eGFR, estimated glomerular filtration rate; HDL, high-density lipoprotein; hsCRP, high sensitivity C reactive protein; IL-34, interleukin; LAd, left atrium diameter; LDL, low-density lipoprotein; LVEDd, left ventricular end-diastolic diameter; LVEF, left ventricular ejection fraction; LVESd, left ventricular end-systolic diameter; NT-proBNP, pro-brain natriuretic peptide; OMI, old myocardial infarction; WBC, white blood cell.*

### Laboratory Examination

Serum levels of IL-34 were measured with fasting venous blood samples, together with NT-proBNP, high sensitivity C-reactive protein (hsCRP), as well as other routine biochemical indices tested at the same time. Once the samples were collected, they were immediately kept on ice and treated within 30 min. To obtain serum, all samples were centrifuged for 20 min at 2000 rpm and stored at –80°C before doing the tests. Human IL-34 Quantikine ELISA Kit (D3400; R&D Systems, Minneapolis, MN, United States) was used to measure the serum levels of IL-34 in obtained samples, according to the manufacturer’s protocol. A modified Modification of Diet in Renal Disease (MDRD) equation was used to calculate estimated glomerular filtration rate (eGFR). The specific formula is as follows: eGFR_MDRD_ = 30849 × serum creatinine (μmol/L) - 1.154 × age - 0.203 [× 0.742 if women] (Levey et al., 2006).

$$\begin{array}{lll} \mathrm{eGFRMDRD}\;=\;30849 & \times & \mathrm{serum}\;\mathrm{creatinine}(\mu \mathrm{mol}/L)\;-\;1.154\\ & \times & \;\mathrm{age}\;-\;0.203[\times \;0.742\;\mathrm{if}\;\mathrm{women}] \end{array}$$

### Coronary Angiography

Coronary angiography was conducted in accordance with the Judkins method and at least 2 vertical angles were observed. All coronary artery lesions were completely exposed and the degree of stenosis was evaluated by quantitative coronary angiography, with the degree of every vascular lesion calculated via Gensini score system (Gensini, 1983).

### Echocardiography

Echocardiography was performed in each subject, using M-mode echocardiography to measure the left ventricular end systolic diameter (LVESD), left ventricular end diastolic diameter (LVEDD), as well as left atrial diameter (LAD). At the end of the breath, the physician drew the left ventricular end-diastolic and end-systolic endocardial contours in the two-dimensional apical four-chamber and two-chamber view with left ventricular ejection fraction (LVEF) automatically calculated by simplified two-plane Simpson’s method.

### Statistical Analysis

Normally distributed continuous variables were expressed as mean ± standard deviation (SD). For those not normally distributed, natural log transformation were performed before analysis. Categorical data were shown as proportions and frequencies. Independent *t*-test or one-way ANOVA test was used to compare continuous variables, while chi-squared test or Fisher’s exact test was performed for the comparison of categorical variables as appropriate. Simple linear regression analysis was used to test the association between variables, along with multivariable linear regression models to determine the relationship between serum IL-34 and NT-proBNP levels. Logistic regression models were used to evaluate the significant predictive value of IL-34 level on the presence of ICM in all subjects and in patients with CAD. Odds ratios (OR) were calculated unadjusted, adjusted for age, sex and body mass index (BMI), and further adjusted for the full model including age, sex, BMI, smoking status, history of hypertension, history of diabetes mellitus (DM), history of dyslipidemia, white blood cell count (WBC), levels of hsCRP, glycosylated hemoglobin (HbA1c), hemoglobin, albumin, low density lipoprotein-cholesterol (LDL-C), eGFR, and medication. IL-34 level was analyzed as a continuous variable with log transformation, as an ordinal variable, and as a categorical variable divided into tertiles. The association of serum IL-34 level with ICM was also analyzed in subgroup studies. SPSS 22.0 (SPSS Inc., Chicago, IL, United States) was used to perform all statistical analyses with a two-tailed *p*-value < 0.05 considered statistically significant.

## Results

### Baseline Characteristics

The present study enrolled a total of 825 subjects, including 360 patients in the ICM group, 232 belonging to the CAD group, along with 233 control subjects without CAD. Patient baseline characteristics and the results of serum biochemistry tests are presented in **Table 1**. Compared to control subjects and to patients with CAD, subjects from the ICM group tended to be older (*P* < 0.001), men (*P* < 0.001), smoker (*P* < 0.001), and drinker (*P* = 0.020); additionally, with more comorbid conditions, for instance, hypertension (*P* = 0.006), DM (*P* < 0.001), as well as CKD (*P* < 0.001), and more frequently a history of stroke (*P* < 0.001). Medication was comparable between CAD patients and those with ICM. However, significantly more patients with CAD and ICM received medication compared to the non-CAD control subjects. The comparisons of HF-defining components, namely, increased serum levels of NT-proBNP, decreased LVEF, as well as increased LVEDD and LVESD, are listed in **Table 1**. As expected, the values of patients with ICM were significantly higher compared to those of patients from both control groups. Moreover, clinical parameters, including BMI, hemoglobin level, platelets count, and albumin level, were remarkably lower in the ICM group, along with higher WBC, creatinine, cystatin C, glucose, troponin I and hsCRP levels, indicating a poorer renal function and general condition, as well as a more severe inflammatory condition in these subjects.

### IL-34 Was Significantly Associated With the Prevalence and Severity of ICM in All Subjects

**FIGURE 1:**
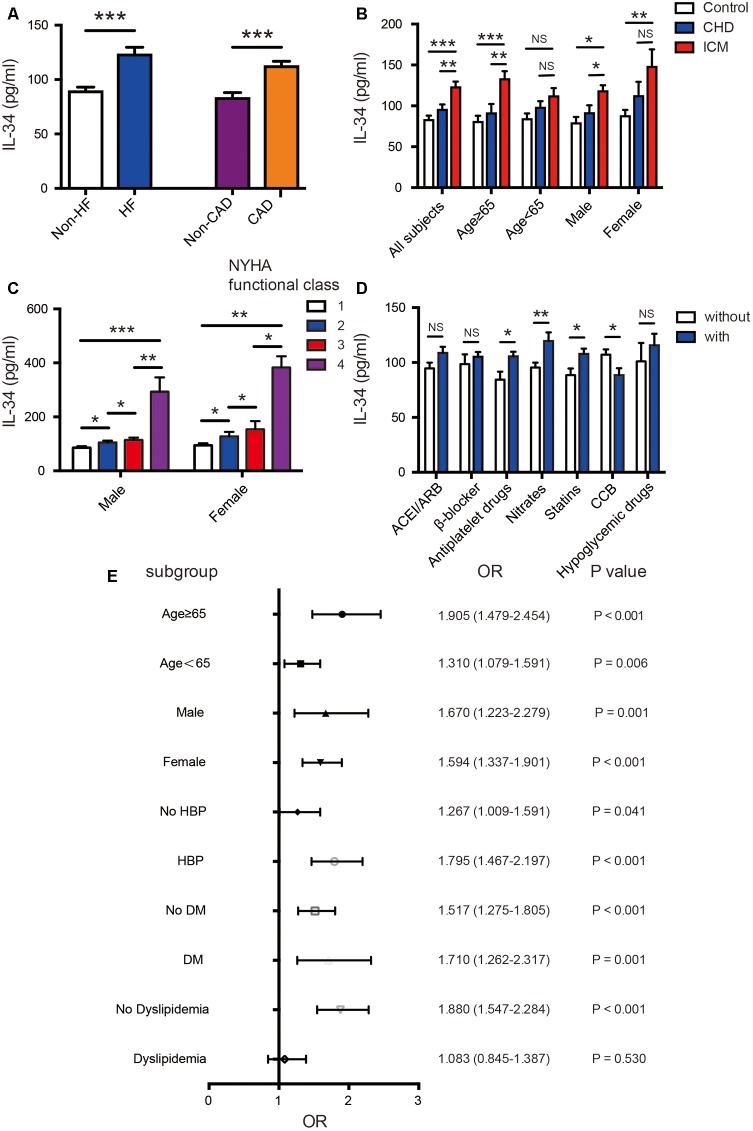
Association of serum IL-34 levels with ischemic cardiomyopathy in all subjects. **(A)** Comparison of serum IL-34 levels between patients with and without heart failure (HF), as well as between those with and without coronary artery disease (CAD), respectively. Among all subjects, serum IL-34 levels were significantly higher in those with HF and in those with CAD. **(B)** Comparison of serum IL-34 levels in all subjects divided into the non-CAD control group, CAD group, and those with ischemic cardiomyopathy (ICM). Stratified analyses were conducted by age (<65 years/≥65 years) and sex (men/women) to determine the relationship between IL-34 level and ICM. **(C)** Serum IL-34 levels were significantly higher in subjects with higher New York Heart Association (NYHA) functional class, and those who belonged to the 4th NYHA functional class showed the highest IL-34 level in both male and female respectively. **(D)** Comparison of serum IL-34 levels in patients using the specific drug and those who didn’t use it, including ACEI/ARB, β-blocker, Antiplatelet drugs, Nitrates, Statins, CCB and Hypoglycemic drugs, separately. **(E)** Forrest plots (unadjusted) to analyze the predictive value of IL-34 level for ICM in different subgroups of all subjects. IL-34 was included as a log transformed continuous variable. NS, not significant; ^∗^*P* < 0.05, ^∗∗^*P* < 0.01, ^∗∗∗^*P* < 0.001.

Serum levels of IL-34 were significantly elevated in patients with ischemic heart failure compared with those without cardiac dysfunction (122.52 ± 136.43 vs. 88.78 ± 93.32 pg/ml, *P* < 0.001); additionally, they were also increased in CAD patients than in those without coronary stenosis (111.75 ± 124.53 vs. 82.57 ± 84.24 pg/ml, *P* < 0.001) (**Figure 1A**). Taking all subjects into 3 groups, patients with ICM tended to have higher IL-34 levels compared to both patients with CAD and normal cardiac function, and to control subjects without CAD (122.52 ± 115.30 vs. 95.02 ± 101.43 vs. 82.57 ± 84.24 pg/ml, respectively; *P* < 0.001) (**Figure 1B**). Furthermore, when subjects were divided in accordance with the New York Heart Association (NYHA) cardiac functional class, those in higher class showed significantly higher levels of serum IL-34 in both males and females separately (**Figure 1C**). A step-by-step increasing tendency remains significant in all subjects (*r* = 0.197, *P* < 0.001), as well as in both males (*r* = 0.274, *P* < 0.001) and females (*r* = 0.241, *P* < 0.001), which indicated that higher levels of IL-34 revealed more severe symptoms of HF regardless of gender.

Simple linear regression analysis indicated that serum levels of log IL-34 were significantly and positively correlated with log NT-proBNP (*r* = 0.223, *P* < 0.001 in all subjects; *r* = 0.240, *P* < 0.001 in male; *r* = 0.210, *P* < 0.001 in female), LVEDD (*r* = 0.209, *P* < 0.001 in all subjects; *r* = 0.209, *P* < 0.001 in male; *r* = 0.216, *P* = 0.002 in female), LVESD (*r* = 0.203, *P* < 0.001 in all subjects; *r* = 0.204, *P* < 0.001 in male; *r* = 0.219, *P* = 0.001 in female), LAD (*r* = 0.178, *P* < 0.001 in all subjects; *r* = 0.207, *P* < 0.001 in male; *r* = 0.146, *P* = 0.035 in female); meanwhile, a negative correlation was detected between log IL-34 levels and LVEF (*r* = –0.190, *P* < 0.001 in all subjects; *r* = –0.198, *P* < 0.001 in male; *r* = –0.212 in female). These results suggested that serum IL-34 levels were significantly correlated with cardiac dysfunction. Multivariable linear regression analysis further verified a significant relationship between IL-34 level and NT-proBNP level (β = 0.171, P < 0.001) after adjusting for age, sex, BMI, history of hypertension, history of DM, and levels of hsCRP, hemoglobin, albumin, HbA1c, as well as eGFR (**Table 2**).

**Table 2:** Univariate and multivariable linear regression models for NT-proBNP in all subjects.

NT-proBNP	Univariate analysis		Multivariable analysis		
	
	β	*P*-value	β	Part.cor.	*P*-value
Log IL-34	0.223	<0.001	0.133	0.171	<0.001
Age	0.325	<0.001	0.185	0.212	<0.001
Gender	0.150	<0.001	0.126	0.161	<0.001
BMI	-0.156	<0.001	-0.102	-0.128	0.001
hsCRP	0.494	<0.001	0.362	0.382	<0.001
Hemoglobin	-0.273	<0.001	–	–	–
Albumin	-0.443	<0.001	-0.153	-0.167	<0.001
HbA1c	0.136	<0.001	–	–	–
eGFR	-0.310	<0.001	-0.127	-0.150	<0.001
HBP	0.175	<0.001	–	–	–
DM	0.148	<0.001	0.133	0.171	<0.001


*IL-34 is analyzed as a log transformed continuous variable. Model: adjusted for age, gender, body mass index, history of hypertension, history of DM, hsCRP, hemoglobin, albumin, HbA1c and eGFR. BMI, body mass index; DM, diabetes mellitus; eGFR, estimated glomerular filtration rate; HBP, high blood pressure; hsCRP, high sensitivity C reactive protein.*

In addition, a lower BMI level was detected in subjects with a higher IL-34 level (*r* = –0.074, *P* = 0.033), together with lower hemoglobin (*r* = –0.168, *P* < 0.001), eGFR (*r* = –0.177, *P* < 0.001), and a higher creatinine (*r* = 0.147, *P* < 0.001) and cystatin C level (*r* = 0.193, *P* < 0.001), suggesting an association between higher IL-34 and poorer renal function, as well as general condition. The relationship remains significant in both male and female subjects.

Furthermore, IL-34 levels were significantly higher in subjects using nitrates (119.60 ± 130.29 vs. 95.46 ± 106.24, *P* = 0.008), antiplatelet drugs (105.50 ± 119.25 vs. 84.44 ± 63.86, *P* = 0.014) and stains (107.84 ± 123.04 vs. 88.52 ± 81.67, *P* = 0.013) compared with those didn’t use. Its levels were also significantly lower in those using CCB treatment (88.41 ± 81.46 vs. 107.17 ± 121.87, *P* = 0.019). Although it’s not quite significant, there’s also an increasing tendency of IL-34 in those who use ACEI/ARB, β-blockers and hypoglycemic drugs (**Figure 1D**). Such correlation was similar in both male and female.

Subsequently, we explored the significant correlation between serum IL-34 level and the risk of prevalent ischemic HF in all studied subjects, taking log-transformed IL-34 as a continuous variable. As demonstrated by univariable and multivariable logistic regression analyses, the association remained significant when unadjusted (OR: 1.549, 95% CI: 1.335–1.798, *P* < 0.001), adjusted for age, sex, as well as BMI (OR: 1.551, 95% CI: 1.327–1.814, *P* < 0.001), and further for the full model (OR: 1.501, 95% CI: 1.249–1.803, *P* < 0.001) including for age, sex, BMI, current smoking, history of hypertension, history of DM, history of dyslipidemia, anti-hypertension medication, anti-DM medication, aspirin and statin use, WBC, and levels of hsCRP, HbA1c, hemoglobin, albumin, LDL-C, eGFR (**Table 3**). To further demonstrate the association between IL-34 level and the presence of ICM, we separately analyzed IL-34 level as an ordinal variable shown in tertiles, and as a categorical variable with the lowest tertile as the reference value. These analyses shed light on the fact that serum IL-34was a significant predictive biomarker for ICM among all subjects, first adjusted for age, sex, and BMI, and then adjusted for further influential factors as described above (**Table 3**).

**Table 3:** Univariate and multivariable logistic regression models for HF.

	Unadjusted OR	*P*-value	Adjusted for Model 1 OR	*P*-value	Adjusted for Model 2 OR	*P*-value
In all subjects						
Log IL-34 per SD	1.549 (1.335–1.798)	<0.001	1.551 (1.327–1.814)	<0.001	1.501 (1.249–1.803)	<0.001
IL-34 tertiles	1.712 (1.438–2.039)	<0.001	1.732 (1.431–2.076)	<0.001	1.521 (1.219–1.898)	<0.001
1st tertile	1 (ref)		1 (ref)		1 (ref)	
2nd tertile	2.366 (1.662–3.367)	<0.001	2.259 (1.562–3.267)	<0.001	1.768 (1.139–2.744)	0.011
3rd tertile	2.987 (2.097–4.253)	<0.001	2.925 (2.085–4.390)	<0.001	2.324 (1.491–3.621)	<0.001
In CAD patients						
Log IL-34 per SD	1.429 (1.198–1.705)	<0.001	1.393 (1.163–1.669)	<0.001	1.391 (1.137–1.702)	<0.001
IL-34 tertiles	1.563 (1.269–1.925)	0.001	1.528 (1.233–1.894)	<0.001	1.417 (1.121–1.791)	<0.001
1st tertile	1 (ref)		1 (ref)		1 (ref)	
2nd tertile	2.274 (1.495–3.457)	<0.001	2.155 (1.405–3.305)	<0.001	1.675 (1.043–2.690)	0.011
3rd tertile	2.475 (1.637–3.742)	<0.001	2.360 (1.542–3.611)	<0.001	2.020 (1.266–3.224)	<0.001


*IL-34 is analyzed as a log transformed continuous variable, an ordinal variable divided according to tertiles of IL-34, and a categorical variable using the lowest tertile as reference. Model 1: adjusted for age, gender, and body mass index. Model 2: adjusted for age, gender, body mass index, smoke, history of hypertension, history of DM, history of dyslipidemia, log WBC, log hsCRP, log HbA1c, hemoglobin, log albumin, LDL-C, eGFR and medication. As a continuous variable, OR is shown as per 1 SD (standard deviation). CAD, coronary artery disease; DM, diabetes mellitus; eGFR, estimated glomerular filtration rate; HF, heart failure; hsCRP, high sensitivity C reactive protein; IL-34, interleukin 34; LDL, low-density lipoprotein; OR, odds ration; WBC, white blood cell.*

On the other hand, in the subgroup analysis, these 3 groups of studied subjects remained significantly different regardless of sex; however, the difference remained significant only in older subjects (aged ≥ 65 years) compared to those aged under 65 years old (**Figure 1B**). The association between IL-34 and NT-proBNP was also more remarkable in the older ones (*r* = 0.262, *P* < 0.001 vs. *r* = 0.175, *P* < 0.001). Moreover, IL-34 levels were more remarkably correlated with echo parameters indicating cardiac dysfunction in those ≥ 65 years old than those <65 (LVEDD: *r* = 0.229, *P* < 0.001 vs. *r* = 0.186, *P* < 0.001; LVESD: *r* = 0.226, *P* < 0.001 vs. *r* = 0.170, *P* < 0.001; LAD: *r* = 0.184, *P* = 0.001 vs. *r* = 0.112, *P* = 0.014; LVEF: *r* = –0.194, *P* < 0.001 vs. *r* = –0.158, *P* = 0.001). Stratified analyses were further conducted to assess the association between ICM and IL-34 level by using a logistic regression model. As shown in **Figure 1E**, serum IL-34 level was a good predictor of worsening cardiac function, especially in older patients, regardless of sex category. The predictive value was also more remarkable in subjects with concomitant diseases such as hypertension and DM, and in those without dyslipidemia.

### IL-34 Was Significantly Correlated With the Presence and Severity of Cardiac Dysfunction in CAD Patients

Since IL-34 levels were significantly elevated in patients with ICM compared with those CAD patients with normal cardiac function, as demonstrated before (**Figure 1B**), we further analyzed the predictive role of serum IL-34 level for cardiac dysfunction especially in those already diagnosed with CAD. Both univariable and multivariable logistic regression models confirmed that IL-34 level was still a significant risk factor for cardiac dysfunction in patients with CAD, including when adjusted for age, sex, BMI, current smoking, history of hypertension, history of DM, history of dyslipidemia, medication, WBC, and levels of hsCRP, HbA1c, hemoglobin, albumin, LDL-C, and eGFR. Separate models were conducted using IL-34 level as a standardized continuous variable with log-transformation, as an ordinal variable, and also as a categorical variable, as described before (**Table 3**).

There’s also a significant relationship between serum IL-34 levels and the Gensini score evaluated by the results of coronary angiography (*r* = 0.189, *P* < 0.001 in all subjects; *r* = 0.193, *P* < 0.001 in male; *r* = 0.245, *P* < 0.001 in female) with simple linear correlation analysis, which indicated that subjects with higher serum IL-34 levels were more likely have severe coronary artery stenosis, possibly leading to more severe ICM. Moreover, we demonstrated that the difference of IL-34 levels between patients with CAD and HF and those with normal cardiac function was more significant in men and in patients older than 65 years (**Figure 1B**).

## Discussion

### Major Findings

Significant associations were determined between serum IL-34 levels and the presence and severity of ICM in all subjects and in patients already diagnosed with CAD, in the present observational study. Notably, multivariate linear regression analysis further demonstrated an independent relationship between serum levels of IL-34 and NT-proBNP. To the best of our knowledge, the present study innovatively detected IL-34 as a novel biomarker of ICM in all subjects and especially in patients with CAD, correlated with cardiac function indexes. IL-34 may take part in the process of ischemic HF via mediating the severity of coronary stenosis, cardiac fibrosis, and inflammatory process; however, the underlying mechanisms remain incompletely understood.

### IL-34 Takes Part in the Process of ICM

Our present study demonstrated that as expected, compared to the control group, subjects from the ICM group tended to be older, men, smoker and drinker, and with more comorbid conditions and medications. They also had more CAD risk factors and clearly, more severe coronary stenosis and renal dysfunction, and significantly poorer general condition and cardiac function. As showed in our results, compared with both the non-CAD control group and CAD group, serum IL-34 levels were significantly elevated in subjects with ICM, correlated with NT-proBNP level, echocardiography parameters, including LVEF, LVEDD, LVESD and LAD, as well as with NYHA functional class, suggesting its function in the process of HF. Additionally, in accordance to the results of previous studies (Li et al., 2012; Fan et al., 2016), our present study have shown that serum levels of IL-34 are significantly elevated in patients with CAD. Furthermore, since ICM is a severe form of end-stage CAD (Chaudhry and Iskandrian, 2005), IL-34 may affect ICM partly through aggravating coronary artery stenosis as it was correlated with the Gensini score. Moreover, as demonstrated the results of our previous study, in the relationship between IL-34 and renal biomarkers, including eGFR and cystatin C (Fan et al., 2016), IL-34 level may take part in the pathophysiological process of HF partly through its effect on renal function. Higher IL-34 level was also associated with poorer general condition, such as anemia and low serum albumin level, leading to more severe cardiac dysfunction. The correlation between medication and IL-34 levels also implied the relationship between IL-34 and the presence and severity of ICM.

Nevertheless, it remains unclear whether IL-34 takes a specific part in the pathogenesis of ICM, or just reflects the presence of cardiac dysfunction in CAD patients. Based on some experimental as well as clinical studies, theoretically speaking, it is rational to speculate that IL-34 has a contributory role in the pathophysiology of ICM, as well as reflects the severity of HF.

### IL-34 Takes Part in ICM by Aggravating CAD

First, IL-34 may be a mediator of inflammatory process in ICM and underlying CAD. In patients with ischemic HF, impaired endothelial function and increased inflammatory process were observed, together with the underlying atherosclerosis participating in this process (Tentolouris et al., 2004; Wilhelmi et al., 2005). Atherosclerosis is a chronic inflammatory reaction with many pro-inflammatory cytokines and chemokines playing important roles in it, mainly through activation of transcription factor NF-κB. Several experiments showed that tumor necrosis factor (TNF)-α can induce IL-34 expression via NF-κB pathway, while IL-34 can act as a pro-inflammatory factor, inducing the secretion of other cytokines and chemokines including IL-6, IL-8, and CCL2 (Eda et al., 2010), and further aggravating the development of atherosclerosis plaque. In addition, macrophage, as an important cell type in the atherosclerosis, is also regulated by IL-34. Since it is capable of up-regulating the expression of chemokine ligands CCL2, CCL4, CCR1, as well as CCR5, which play a part in the recruitment of monocytesand macrophages (Preisser et al., 2014), IL-34 may influence the process of ischemic myocardial injury and atherosclerosis by regulating monocytes migration and macrophages differentiation (Swirski et al., 2009; Robbins et al., 2012), as well as mononuclear phagocyte adhesion to the endothelium and angiogenesis (Segaliny et al., 2015).

It is likely that IL-34 may be an influential factor of CAD and ICM as a pro-inflammatory cytokine, inducing the secretion of other pro-inflammatory cytokines (Masteller and Wong, 2014) and the accumulation of macrophages, further aggravating the process of CAD (Swirski et al., 2009) and, ultimately, worsening the HF.

### IL-34 Takes Part in ICM via Mediating Cardiac Remodeling

Second, the significant association of IL-34 level with parameters indicating cardiac enlargement and systolic dysfunction suggested that IL-34 might be a mediator of cardiac remodeling in ICM. It has been demonstrated that cardiac remodeling in ICM is associated with inflammation, interstitial fibrosis, and ventricular dysfunction (Frangogiannis, 2004). Chemokine expression and inflammation status play essential roles in the process of ICM, affecting several genes associated with cardiac fibrosis and cardiac remodeling (Gronich et al., 2010). So that IL-34 may affect ischemic myocardial injury through regulating inflammation and innate immunity (Eda et al., 2010).

On the other hand, IL-34 has been found overexpressed in chronic hepatitis, inducing pro-fibrotic macrophages to release transforming growth factor β, platelet-derived growth factor, as well as galectin-3 (Preisser et al., 2014). Galectin-3 is a well-known biomarker of cardiac remodeling and HF (Ho et al., 2012; Bayes-Genis et al., 2014; Wu et al., 2015). Plenty of researches demonstrated that higher concentration of galectin-3, as a biomarker of cardiac fibrosis, was significantly correlated with increasing risk of incident HF and all-cause death in the community. Moreover, it could predict poor outcomes, including cardiovascular death, in patients with HF (Bayes-Genis et al., 2014). What is more important, transforming growth factor β was also found involved in the transition from inflammation to fibrosis in ICM (Frangogiannis, 2004). Taking into consideration that IL-34 level was significantly elevated in ICM patients in comparison with those already diagnosed with CAD, but with normal cardiac function, along with the contribution of IL-34 as a risk factor of HF in patients with CAD, it may exacerbate cardiac dysfunction as a potent pro-fibrotic factor inducing cardiac fibrosis through galectin-3 and transforming growth factor β pathway., Nevertheless, it’s still of vital importance to further explore the direct function of IL-34 in cardiac remodeling and its underlying mechanisms.

### IL-34 Takes Part in ICM Through the Influence on Renal Function

Our previous study demonstrated the predictive role of IL-34 on renal dysfunction during HF (Fan et al., 2016). We also found that serum IL-34 levels were significantly associated with poor prognosis in patients with chronic heart failure, especially in those with renal impairment (Tao et al., 2017). Previous basic experiments on the kidney also showed that compared to IL-34–/– mice, more tubular pathology and leukocytes were detected in the interstitium after I/R injury, and more severe renal fibrosis was found in the chronic phase in wild-type mice (Baek et al., 2015). Therefore, IL-34 might deteriorate kidney function in ICM, which could in turn affect cardiac function, resulting in a vicious circle.

To sum up, all above results suggested that IL-34 might take part in the process of ischemic HF through inflammation response and fibrosis progress, aggravating CAD, cardiac remodeling, renal function, as well as patient general condition in ICM. It may also be a consequence of ICM and serve as a biomarker.

### Study Limitations

The present study novelly demonstrated the relationship between serum IL-34 and the presence and severity of ICM, which is the major cause of heart failure, and also expanded the sample size in all subjects including the controls and especially in CAD patients, thus contributing to the current literature., Nonetheless, the present study has several limitations. First, no causal inference could be discerned since it was a cross-sectional study. So that the relationship between serum IL-34 level and the risk of cardiac dysfunction, especially in patients with CAD but without heart failure at baseline, needs to be illustrated in larger cohorts to prospectively confirm the predictive value of it. Also new tools such as global longitudinal strain are needed to evaluate cardiac function more accurately, further confirming the association between IL-34 and heart failure. Second, although we expanded the study group and determined the impact of IL-34, future studies on larger cohorts including subjects with various environmental as well as genetic backgrounds are needed to verify our findings. Since the presented results only shows the correlations, more researches are warranted to demonstrate the role of IL-34 during ICM, for example, as a driver, an influential factor or a consequence. Thus, further studies are necessary so as to better explore the underlying mechanism of the IL-34 influence during HF, particularly in ICM. In addition, serum IL-34 level could probably be influenced by other unknown confounders or undetermined comorbidities, including some influential factors which could affect cardiac function or general condition, taking part in the process of atherosclerosis or cardiac remodeling in ICM. Several variables still need to be determined and further analyzed despite of the confounding factors for which we have adjusted in the present study.

## Conclusion

To conclude, the present study determined that serum IL-34 levels were remarkably elevated in ICM patients, significantly correlated with the presence and severity of ischemic HF. In addition, IL-34 levels were significantly associated with cardiac dysfunction parameters, such as NT-proBNP level, and cardiac echocardiography indices. These results suggest that IL-34 may involved in the process of ischemic HF and be used as a novel biomarker and clinical predictor of ICM in the selected population, and especially in those with CAD.

## Author Contributions

All co-authors have made critical contributions to the present study, including study design, data collection, analyses and interpretation as well as preparing the paper itself. They also have read and approved the final version. In detail, RX, QF, FW, and RT contributed to all aspects consisting of the conception and study design, along with the acquisition, analysis and explanation of the data. RX and QF contributed to drafting the manuscript, together with FW and RT taking part in revising and approval of the final version before submission. XY, HZ, and HX were integral to the study design and data collection. GG and YX assisted with data analysis and interpretation, along with revising the manuscript.

## Conflict of Interest Statement

The authors declare that the research was conducted in the absence of any commercial or financial relationships that could be construed as a potential conflict of interest.
